# Revealing the Intrinsic Restructuring of Bi_2_O_3_ Nanoparticles into Bi Nanosheets during Electrochemical CO_2_ Reduction

**DOI:** 10.1021/acsami.3c18285

**Published:** 2024-02-26

**Authors:** Beatriz Ávila-Bolívar, Mauricio Lopez Luna, Fengli Yang, Aram Yoon, Vicente Montiel, José Solla-Gullón, See Wee Chee, Beatriz Roldan Cuenya

**Affiliations:** † Institute of Electrochemistry, University of Alicante, Alicante 03690, Spain; ‡ Department of Interface Science, Fritz Haber Institute of the Max Planck Society, Berlin 14195, Germany

**Keywords:** bismuth oxide nanoparticles, carbon dioxide electroreduction, catalyst restructuring, *in situ* studies, liquid cell transmission electron microscopy, *operando* Raman spectroscopy

## Abstract

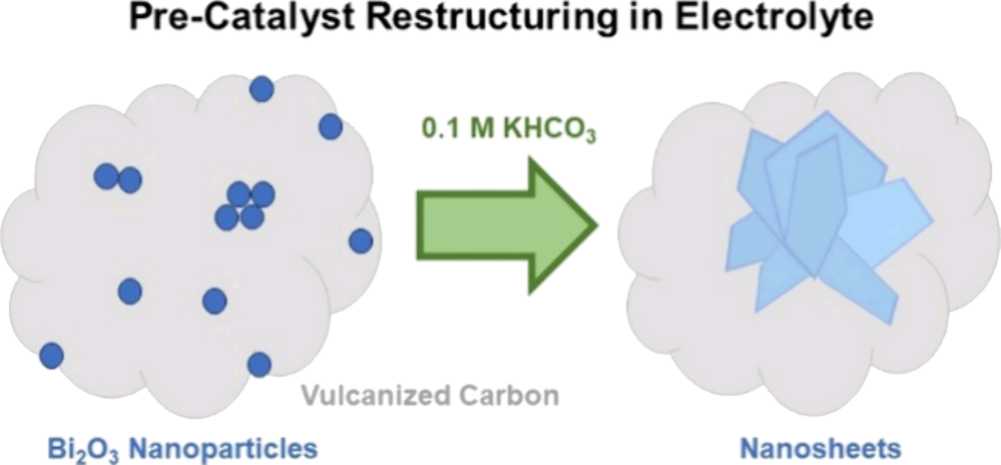

Bismuth is a catalyst material that selectively produces formate during the electrochemical reduction of CO_2_. While different synthesis strategies have been employed to create electrocatalysts with better performance, the restructuring of bismuth precatalysts during the reaction has also been previously reported. The mechanism behind the change has, however, remained unclear. Here, we show that Bi_2_O_3_ nanoparticles supported on Vulcan carbon intrinsically transform into stellated nanosheet aggregates upon exposure to an electrolyte. Liquid cell transmission electron microscopy observations first revealed the gradual restructuring of the nanoparticles into nanosheets in the presence of 0.1 M KHCO_3_ without an applied potential. Our experiments also associated the restructuring with solubility of bismuth in the electrolyte. While the consequent agglomerates were stable under moderate negative potentials (−0.3 V_RHE_), they dissolved over time at larger negative potentials (−0.4 and −0.5 V_RHE_). *Operando* Raman spectra collected during the reaction showed that under an applied potential, the oxide particles reduced to metallic bismuth, thereby confirming the metal as the working phase for producing formate. These results inform us about the working morphology of these electrocatalysts and their formation and degradation mechanisms.

## Introduction

The electrochemical carbon dioxide reduction reaction (CO_2_RR) is a promising route to reduce the amount of CO_2_ released in the atmosphere,^1−3^ whose increase is a key reason for the rise in global temperatures and its damaging consequences.^4−9^ CO_2_ is a stable molecule, and rationally designed efficient electrocatalysts are necessary for its reduction.^10^ However, despite extensive efforts to synthesize different precatalysts to optimize the catalytic performance, the lack of understanding into how electrocatalysts evolve under working conditions has hampered our ability to engineer more active catalysts that are also stable during long-term operation. In this context, *in situ* and *operando* microscopy and spectroscopy methods can provide valuable insight into the actual working state of the catalysts.^11,12^

Bismuth nanoparticles (NPs) have been widely studied as a catalyst for CO_2_RR^13−20^ because bismuth is highly selective toward formate production.^21−23^ Previously, we developed Bi_2_O_3_ NPs supported on vulcanized carbon (Bi/C) that showed very high activity and selectivity.^14,16^ To explore the practical application of these catalysts, the samples were also tested in a commercial flow cell working in continuous mode, where the input to the cathode was only humidified CO_2_, without a catholyte.^16^ The measurements showed one of the best balances between the formate concentration, Faradaic efficiency, and energy consumption. The poor long-term stability of these Bi/C NPs in both H-type cell and flow cell reactors is, however, detrimental to their use in an industrial setting. Alternatively, nanosheets (NSs), another commonly synthesized form of bismuth electrocatalysts, have been reported to exhibit superior performance for CO_2_RR.^20,24−29^ Interestingly, it was also described that Bi_2_O_3_ NPs can transform into 2D nanosheets after 20 linear sweep voltammetry cycles at 0 to −1.3 V versus RHE.^30^ Since only the morphology before and after the reaction was characterized in this study, it was unclear whether the evolution of the Bi_2_O_3_ NPs occurred at specific junctures of the electrocatalytic protocol or if it was a more general phenomenon. Recent studies using *operando* Raman spectroscopy have also shown the activation of Bi surfaces due to an electrolyte-mediated structural transformation^31^ where the Bi–O band only appeared in the presence of an electrolyte. In line with the electrolyte dependence for bismuth-based catalysts, the evolution of bismuth oxide nanowires into larger ultrathin bismuth nanosheets covered with an amorphous oxide thin layer was also reported.^32^

In this work, we studied the morphological evolution of Bi/C NPs using liquid cell transmission electron microscopy (LC-TEM) and *operando* Raman spectroscopy to better understand their nanoscale morphologies within the electrolyte and under CO_2_RR conditions. LC-TEM has been successfully used previously to study *in situ* the transformations of CO_2_RR electrocatalysts under potential control.^33−36^ Here, we used both *ex situ* and *in situ* TEM to characterize the Bi_2_O_3_ NPs before, during, and after the reaction, which describes drastic restructuring of the NPs once they were in contact with the 0.1 M KHCO_3_ electrolyte. *Operando* Raman spectroscopy experiments were further performed to confirm the oxidation state of Bi/C NPs during the CO_2_RR.

## Experimental Section

### Synthesis and *Ex Situ* Characterization of Bi/C NPs

The Bi/C NPs were synthesized at room temperature and atmospheric pressure following the same methods that we described in previous works.^14,16^ Specifically, 0.316 g of BiCl_3_ was dissolved into 37.92 g of DMF, and then, 0.112 g of PVP K30 was added and sonicated until complete solubilization was achieved. For the chemical reduction of Bi^3+^, 0.116 g of NaBH_4_ was added. The solution was stirred by alternating magnetic stirring and ultrasonication (each 10 min) for 45 min. Afterward, carbon Vulcan XC-72R powder was mixed with the Bi NPs to obtain a Bi loading of ca. 20 wt %. After another 90 min of magnetic stirring and ultrasonication, Bi/C NPs were precipitated by using acetone (about 5 times the DMF volume), and this was repeated several times. The samples were cleaned by washing them with a mixture of acetone/water (80/20). Finally, the NPs were dried overnight in vacuum at 55 °C. In order to evaluate the effect of the pH on the Bi NPs, the test was conducted where the Bi/C NPs were alternatively washed with 100 mL of 0.5 M NaOH solution during the filtering process.

Figure S1 shows TEM images of the freshly synthesized Bi/C NPs. The particle size, morphology, and dispersion of the Bi_2_O_3_ nanoparticles on the carbon support were similar to those that we reported previously.^14^ The quasi-spherical NPs had a mean particle size of about 10 nm and were homogeneously dispersed on the Vulcan carbon. As we had reported previously,^14,16^ these samples are highly selective toward the production of >90% in KHCO_3_ electrolytes with hydrogen found as the other minor subproduct.

### *Ex Situ* Transmission Electron Microscopy

TEM images were first acquired with a JEOL JEM-1400 Plus working at 120 kV. For imaging, the as-prepared samples were dispersed in ethanol and supported on a TEM grid. These images verify the size, morphology, and dispersion of the Bi/C NPs. High-resolution (HR)-TEM images were taken from the samples before and after the electrocatalytic experiments with a Titan 80-300 at 300 kV (Thermo Fisher Scientific). Energy-dispersive X-ray analysis (EDX) elemental maps were acquired using a 200 kV Talos TEM (Thermo Fisher Scientific).

### *In Situ* Transmission Electron Microscopy

The LC-TEM experiments were performed using a Hummingbird Scientific bulk liquid electrochemistry TEM holder in the Titan TEM at 300 kV and in the STEM mode. The holder was equipped with customized Ag/AgCl reference and Pt counter electrodes. The electrochemical liquid cell chips with a carbon working electrode were also manufactured by Hummingbird Scientific. The Bi/C NPs were dispersed in ethanol, and a drop of ∼20 μL was deposited on the TEM chip, leaving it to air-dry at room temperature, after which the full cell was assembled.

After cell assembly, the TEM holder was prefilled with Milli-Q water for leak checking before it was introduced into the TEM. After loading into the TEM, the syringe was first filled with 0.1 M KHCO_3_ or with CO_2_-saturated 0.1 M KHCO_3_ solution for the CO_2_RR experiments. Our saturation procedure involved bubbling CO_2_ gas through the 0.1 M KHCO_3_ solution for 30 min before aliquots of the freshly saturated solution were extracted. The electrolyte was then injected into the holder using a syringe pump. The flow rate used was 1.5 mL min^–1^.

The CO_2_RR experiments were performed in a three-electrode geometry with a Pt mesh as counter and AgCl/Ag (3 M KCl) as reference electrodes. The potentials were then converted to the RHE scale using Nernst’s equation. The pH of the carbon dioxide-saturated electrolyte was 6.8. The electrochemistry experiments were controlled by a Biologic SP-200 potentiostat. Cyclic voltammetry from 0.2 to −0.5 V_RHE_ was carried out to determine the most negative potential where we did not see bubble formation due to HER. Chronoamperometry measurements were performed during 1 h at −0.3, −0.4, and −0.5 V_RHE_. The current intensity was normalized by the geometric area of the chip window to obtain the corresponding current density (*j*/mA cm^–2^).

### *Operando* Raman Spectroscopy

First, a working electrode was prepared by air-brushing and dispersing a catalytic ink (Bi/C NPs, Nafion solution, and isopropanol) on a 1 × 1 cm^2^ Toray paper that is supported on a hot metallic plate at ∼90 °C to facilitate solvent evaporation. The Bi loading was 0.1 mg cm^–2^. *Operando* Raman spectroscopy was performed using a confocal Raman spectrometer (Renishaw, InVia Reflex). Three different lasers were used as excitation sources (λ = 532, 633, and 785 nm). The backscattered light was Rayleigh-filtered and collected in the range of 20–3700 cm^–1^ using a grating of 1600 lines mm^–1^. Before each experiment, the spectrometer was calibrated using a Si(100) wafer (520.5 cm^–1^). The *operando* cell used in the experiments consisted of a single compartment made of PTFE and equipped with a leak-free Ag/AgCl reference electrode and a Pt wire as a counter electrode. A freshly CO_2_-saturated 0.1 M KHCO_3_ solution was used as an electrolyte. The laser exposure and Raman scattering results were collected using a water immersion objective (Leica Microsystems, 63×, NA 0.9). The water immersion objective was protected from the electrochemical environment by a Teflon film (DuPont, 0.013 mm thickness). During the constant potential experiments, the electrode was kept at the potential indicated for 5 min before spectrum collection.

## Results and Discussion

### *In Situ* Observations of Bi/C NP Transformations during Contact with the Electrolyte

**Figure 1:**
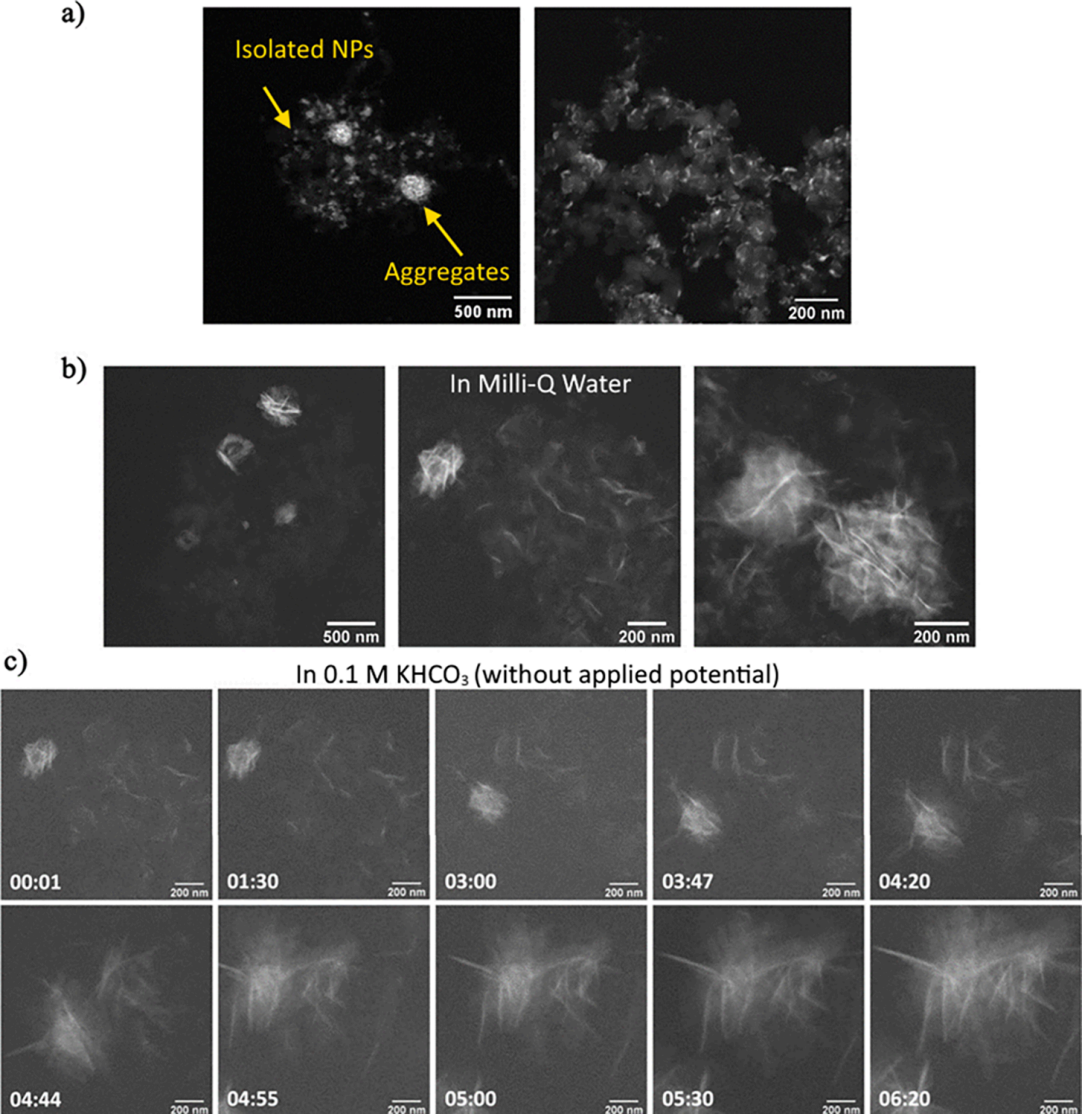
(a) STEM images of the carbon-supported Bi_2_O_3_ NPs dispersed on the working electrode of an LC-TEM chip before the assembly at different magnifications, (b) LC-TEM images of the sample with only Milli-Q water flow at open-circuit potential illustrating nanosheet aggregation under this condition, and (c) LC-TEM images that were collected while the samples were in contact with 0.1 M KHCO_3_ solution at open-circuit potential showing extended restructuring into nanosheets. The time stamps are in minutes:seconds.

Figure 1 shows TEM images collected *ex situ* and *in situ* during our experiments where *in situ* LC-TEM experiments were performed under similar conditions to our *ex situ* H-type cell setup. First, the chip electrode was analyzed by high-angle annular dark-field scanning TEM (STEM) before assembly to characterize the NP arrangement (Figure 1a). The images obtained were similar to those that we reported previously with TEM^16^ (Figure S1) where the Bi/C NPs now appear bright in the image due to the *Z*-contrast. Larger agglomerations of NPs can be also seen in the precatalysts. The liquid cell was then assembled, and the LC-TEM holder was prefilled with Milli-Q water before being inserted into the TEM. The resolution and contrast of the images when the samples were flooded with liquid were lower,^37^ and so, the isolated NPs could no longer be resolved in the images, but the NP aggregates could be seen (see Figure 1b). It appeared, however, that the NPs had already undergone some restructuring. Next, we continued to track the sample as unsaturated 0.1 M KHCO_3_ solution was introduced via the fluid tubing. Figure 1c shows the structural changes in the Bi/C NPs over time under the flow of the electrolyte solution. The first images showed sheetlike structures around 200 nm in size that could be dispersed or agglomerated into sphere-shaped objects. Since the sample was immersed in Milli-Q water, these initial changes are presumably caused by the liquid immersion. The sample remained unchanged until about the third minute where the bright bismuth structures started to move and join to form longer nanosheets and larger agglomerated star-shaped structures. This transformation takes place over approximately 2 min. After 5 min, no further change is observed. A comparison between the initial and final shape at a higher resolution is provided in Figure S2. To ensure that this is not an electron beam-induced effect, we checked the sample at a lower magnification and found similar structures in other regions around the imaged area (Figure S3). The NSs had sizes between 400 and 700 nm, and the agglomerated structures could be up to 1 μm in diameter. While most of the Bi oxide NPs were transformed into NSs, we could still find some isolated NPs (Figure S3d), presumably due to them being deeply embedded in the support and not exposed to the electrolyte.

**Figure 2:**
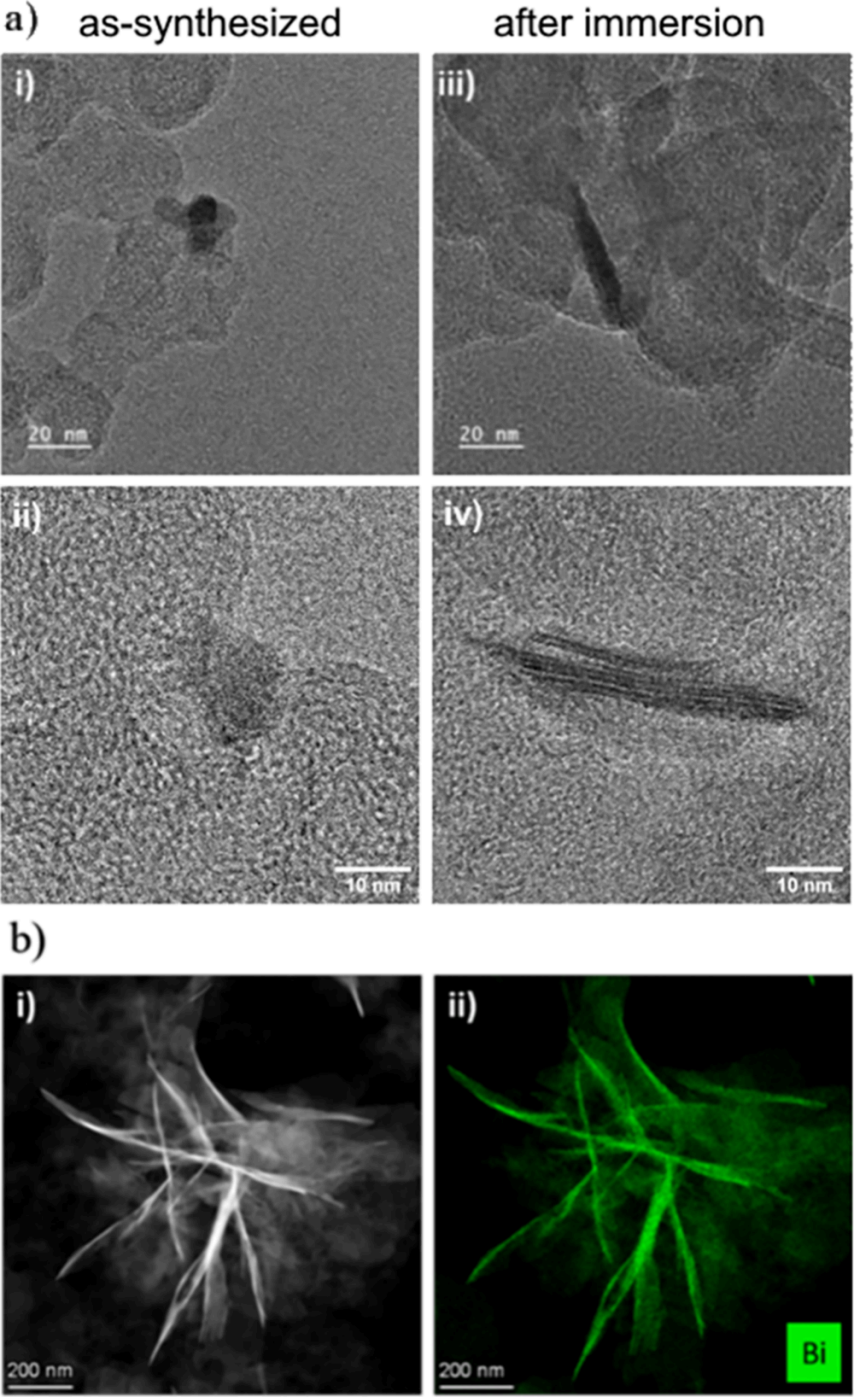
(a) HR-TEM images of the carbon-supported Bi_2_O_3_ NPs (i,ii) before and (iii,iv) after contact with 1 M KHCO_3_ solution and (b) EDX-TEM images of samples on the LC-TEM chip after the CO_2_RR reaction in CO_2_-saturated 0.1 M KHCO_3_ during 1 h at different potentials where (i) is the STEM image and (ii) is the bismuth EDX map.

*Ex situ* analysis of the samples on the LC-STEM chip was also performed to better understand the structural and chemical changes that occurred. Figure 2a compares the NPs and the nanosheets with high-resolution TEM (additional images can be found in Figures S4 and S5 for NPs and NSs, respectively). EDX mapping shown in Figure 2b confirms that the nanosheets consist of Bi. Figures S6 and S7 show the overall chemical composition for these structures.

Lee et al.^38^ had previously discussed the transformation of Bi_2_O_3_ particles into bismuth nanosheets during voltammetric cycling in a CO_2_-saturated 0.5 M KHCO_3_ electrolyte. They explained this transformation as the exfoliation of bismuth layers from the Bi_2_O_3_particles. This hypothesis is, however, inconsistent with our *in situ* observations. Our images show the nanosheets forming in the electrolyte at open-circuit potential (OCP). We reiterate that the samples/reaction cells in our experiments were introduced into the TEM with water in the cell but without an applied potential. Therefore, the structural change toward nanosheets was not due to the electrochemical reduction of Bi_2_O_3_ to bismuth metal. Instead, we speculate that the nanosheet formation may be intrinsic to bismuth-based catalysts and that the extended transformation is caused by the chemical conditions created during electrolysis based on the fact that previous work^30,38^ had generally reported similar stellated or nanosheet structures at ambient pressure conditions after CO_2_RR.

First, we consider whether the structural changes reported in previous work may be explained by the pH of the electrolyte where an increase in local pH can arise from the consumption of protons by CO_2_RR and HER.^39−41^ For one, we found that the restructuring accelerated when we switched the electrolyte from water to unsaturated KHCO_3_ under OCP, which has a pH of 8.3. To verify this hypothesis, we rinsed the as-synthesized Bi/C with a 0.5 M NaOH solution (pH ≈ 14, no applied potential) instead of the water/acetone mixture (pH ≈ 7) used in our standard washing procedure. As shown in Figure S8a, the nanosheets were again created under these alkaline conditions. The size of these structures is very varied, around 30–200 nm. Here, the stability of the NPs during washing with the water/acetone mixture is likely due to the high acetone content as we have shown that the catalysts restructure moderately in Milli-Q water (Figure 1b). We also note the consistency of our results with previous synthesis work where Bi_2_O_3_ nanoparticles^42−45^ synthesized at pH of 9 or 10 became transformed into larger spherical blocks,^42^ longer platelets,^43^ nanoparticle aggregate blocks,^44^ and large-sized nanosheets^45^ when the solution pH is raised.

**Figure 3:**
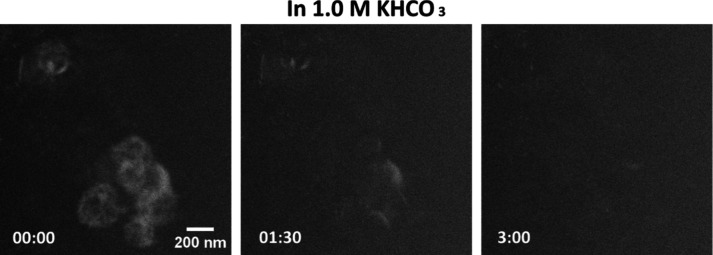
STEM image sequence collected as 1.0 M KHCO_3_ solution was introduced into the LC-TEM cell at an open-circuit potential showing bismuth dissolution. The time stamps are in minutes:seconds.

Therefore, the precatalyst restructuring appears to be associated with the electrolyte. To further check for the effect of the electrolyte, we performed LC-TEM studies of these samples in 1.0 M KHCO_3_ solution. As shown in Figure 3, our *in situ* observations at OCP reveal no nanosheet formation but extensive bismuth dissolution in the more concentrated electrolyte instead. Bismuth dissolution under such conditions was also verified with *ex situ* comparisons of the samples drop casted on a TEM grid with a holey carbon support before and after 10 min of immersion in 1.0 M KHCO_3_ (Figure S8b). Based on these results, we can now attribute the precatalyst evolution with the solubility of Bi_2_O_3_ in the electrolyte. In 0.1 M KHCO_3_, the moderate solubility of bismuth enables mass transport of bismuth while also allowing for reprecipitation, which facilitates the transformation into nanosheets. Conversely, dissolution becomes increasingly favored as the solubility of bismuth increases with the concentration of the electrolyte.

### *In Situ* Observation of Bi Nanostructures during CO_2_RR

To understand the stability of the nanosheet structures during CO_2_RR, we performed LC-TEM experiments under constant potential (chronoamperometry). First, the 0.1 M KHCO_3_electrolyte was saturated with CO_2_, and then, the solution was circulated through the cell during 15 min at OCP. No change in the Bi stellate structures was observed during this time. Cyclic voltammetry (CV) was next carried out to determine the potential for CO_2_RR (Figure S9), which showed a reduction process starting at −0.2 V_RHE_. However, we started to observe persistent bubble formation when the potential was decreased to −0.6 V_RHE_, which we attribute to the competing hydrogen evolution reaction (HER). These bubbles terminated further *in situ* experiments as they washed the samples away from the working electrode. Therefore, we were limited to CVs in the range from 0.2 to −0.5 V_RHE_, and we were unable to reach the potential of −1.0 V_RHE_ where CO_2_RR on Bi usually reaches its optimum performance.

**Figure 4:**
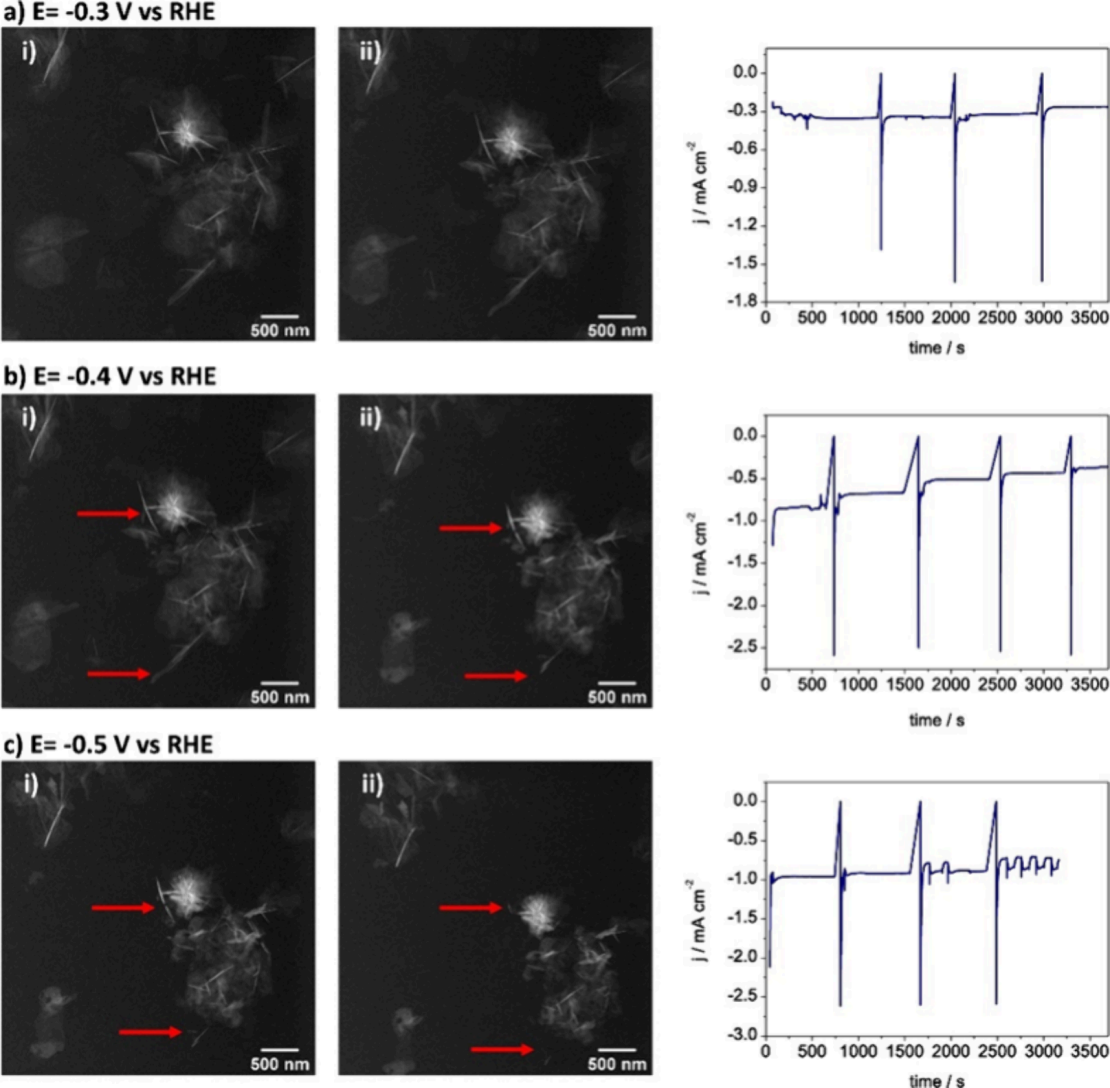
(i) Initial and (ii) after 1 h LC-TEM images of the electrocatalysts at constant applied potentials of (a) −0.3, (b) −0.4, and (c) −0.5 V_RHE_ in CO_2_-saturated 1 M KHCO_3_ solution. The current jumps seen every 15–20 min are due to the pause in the chronoamperometry when the electrolyte-containing syringe is exchanged to maintain the electrolyte flow. At *E* = −0.5 V_RHE_, the current density started to oscillate abruptly from about minute 40 onward. These irregularities in the current may be due to the formation of hydrogen microbubbles, which are not observed in the imaged area but may exist in other parts of the electrode. Red arrows highlight examples where nanosheet dissolution is seen.

Next, we followed the stellate structures during ECO_2_RR with chronoamperometry for 1 h at different applied potentials. Figure 4 shows the initial and final state of the sample at −0.3, −0.4, and −0.5 V_RHE_ after 1 h. In general, we see decreasing stability of the stellate structures as we go from −0.3 to −0.5 V RHE, where more severe dissolution was seen at the more negative potentials. Despite the lower overpotential of these observations, catalyst dissolution is clearly the primarily degradative mechanism for these electrocatalysts.

Therefore, these results provide clues into the mechanisms of degradation in the early stages of CO_2_RR. While the stellate structures largely remain unaltered at potentials where we see the reduction of Bi_2_O_3_to the metallic state (−0.3 V_RHE_), the stability of these structures is clearly compromised when more negative potentials are applied. Although there are different mechanisms of catalyst degradation,^46^ the images acquired during chronoamperometry (Figure 4) suggest that in the present example, the catalysts primarily undergo dissolution or material detachment due to the formation of small hydrogen bubbles during the reaction.

### *Operando* Raman Spectroscopy of Bi/C NPs during the ECO_2_RR

*Operando* Raman spectroscopy experiments were also carried out to probe the structural and compositional changes in the sample during the reaction. The sample was analyzed with the three lasers available in the spectrometer at *ex situ* conditions (λ = 532, 633, and 785 nm). As can be seen in Figure S10, the spectra are very similar for the three lasers. The dominant features observed in the *ex situ* spectra correspond to the carbon Vulcan powder (the first-order G band at 1316–1341 cm^–1^ and the D band at 1589–1602 cm^–1^).^47,48^ No other visible signals attributed to any metallic Bi state were observed.

**Figure 5:**
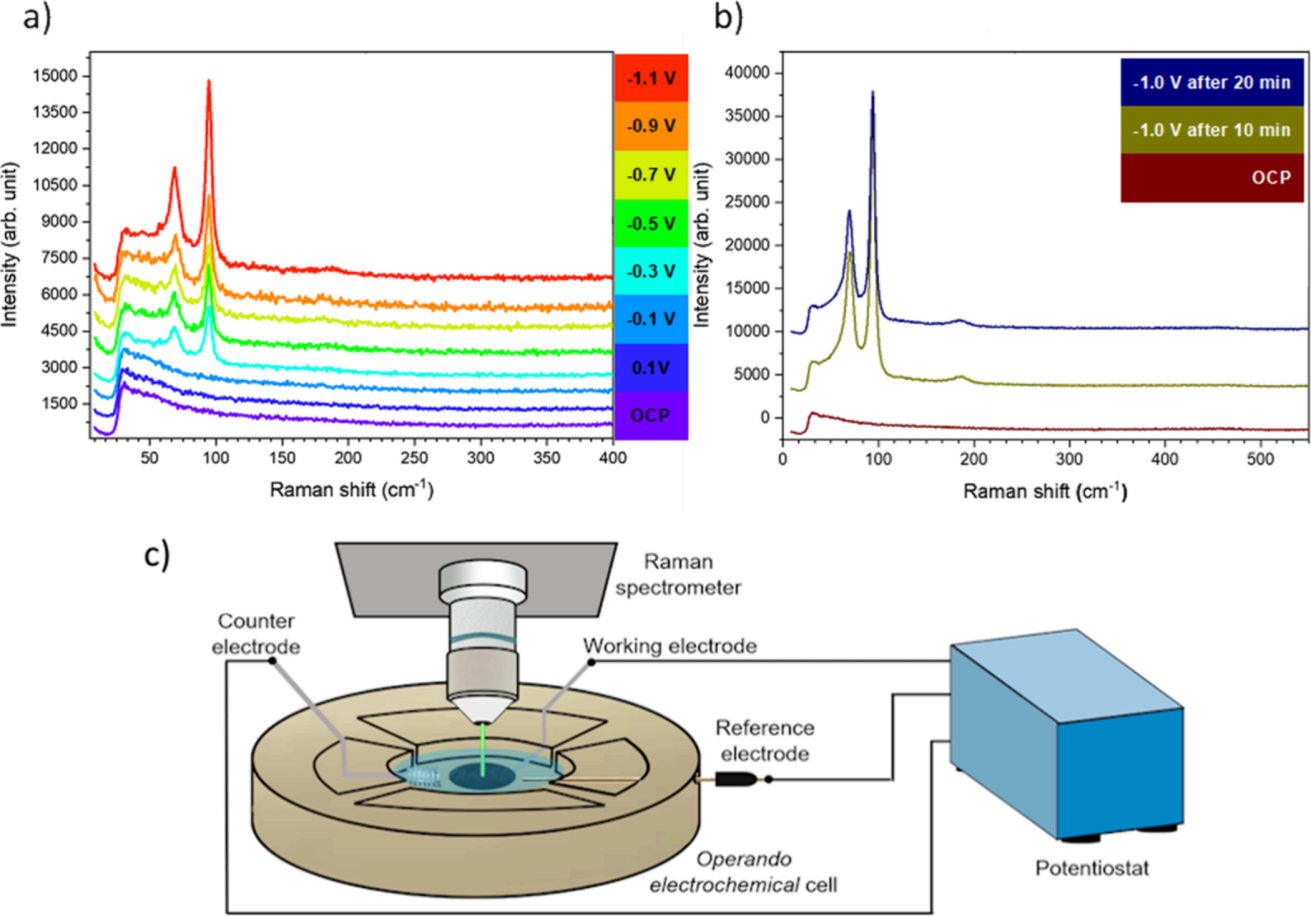
*Operando* Raman spectra of the Bi/C samples collected with a λ = 785 nm laser (a) at different constant potentials and (b) at OCP and at −1.0 V_RHE_ over time. The spectra are offset in the *y*-direction for better visualization. (c) Schematic of the *operando* Raman spectrometer setup.

Figure 5a shows the *in situ* Raman spectra acquired using a λ = 785 nm laser during stepwise incremental potential application. Starting at −0.3 V_RHE_, two new bands (at about 70 and 94 cm^–1^) appeared on the low Raman shift region. The normalized intensity of these two bands also increased as the potential became more negative (in Figure S11a, the spectra are normalized to the intensity of the edge of the notch filter cutoff). According to the literature, these bands correspond to the doubly degenerated E_g_ and nondegenerated A_1g_ phonon modes (70 and 92 cm^–1^, respectively) of rhombohedral metallic bismuth.^30,49^ Here, the metallic bismuth Raman peaks appear at 70 and 94 cm^–1^, which indicates a small downshift compared to the expected values^30,49^ and could be a consequence of phonon confinement in these nanometer-sized Bi samples.^50^ In addition, metallic bismuth modes become very well-defined at −0.4 V_RHE_, and a small feature shows up at 185 cm^–1^ that appears more visible at more negative potentials. Previous work suggested that the band at 185 cm^–1^ corresponds to the stretching mode of Bi–O together with a band at about 98 cm^–1^.^30,49^ However, such an assignment is unlikely in our case since as it is shown in the spectra taken at OCP, this mode is not found in our Bi/C precatalyst NPs, which should have had the highest content of oxidic Bi species. The absence of signals from the oxide signatures in the as-prepared samples is likely due to the poor crystallinity of the starting Bi oxides, and so, this new mode is not a signature of residual oxides (Table S1 contains detailed Raman peak positions for different Bi oxides). To complete this analysis, Figure 5b shows the comparison between the initial state (OCP conditions) and that after applying a negative potential of −1.0 V_RHE_ for different periods of time. It is worth noting that after applying the negative potential, the spectra remain essentially unaltered, which points out the stability of the sample under these electrochemical working conditions. In addition, the band at 185 cm^–1^ persists during the reaction, as shown in Figure S11b with the normalized spectra at OCP and at a 20 min reaction time and does not decrease with time. Thus, the band at 185 cm^–1^ is more reasonably assigned the overtone of the metallic bismuth phonon modes,^51^ which is in line with the earlier observations of the metallic phase under negative potentials by Feng et al.^52^ Therefore, our*operando* Raman results indicate that the NPs electrochemically reduce to metallic bismuth at negative potentials below −0.3 V_RHE_.

The high Raman shift frequency regime was also inspected with the 633 nm laser to study intermediate states during the reaction (Figure S12). Under this configuration, the peak at 70 cm^–1^ is not observed because of the notch filter cutoff of the optics. Again, the gradual increase of the Bi metallic band at about 94 cm^–1^ and the overtone at about 185 cm^–1^ are clearly observed. Moreover, the G and D bands, together with the second-order G and D bands ( ∼2800 and ∼3400 cm^–1^) from the carbon substrate, do not exhibit any relevant perturbation under electrochemical conditions. According to the Raman spectra, no further changes in the chemical state of the sample are detected once the sample is electrochemically transformed into metallic bismuth below −0.3 V, regardless of the applied potential and time.

## Conclusions

Here, our work highlights three key aspects regarding the behavior of Bi/C NPs during CO_2_RR, (i) the restructuring of the catalysts upon exposure to the electrolyte, (ii) the degradation of the catalysts under an applied potential, and (iii) the active phase. LC-TEM studies indicate that Bi/C NPs undergo restructuring into stellated nanosheet agglomerates as soon as they are immersed in the electrolyte (i.e., at OCP). The restructuring is explained by the solubility of bismuth in the electrolyte. We further show that these structures undergo slow degradation under sustained reaction conditions. Our *operando* Raman studies unveiled that the NPs are reduced to metal bismuth at moderately negative potentials. More importantly, these observations suggest that the NP-to-nanosheet transformation is intrinsic to reactions under typical CO_2_ reduction reaction conditions. Our *in situ* observations highlight how restructuring due to the electrolyte or the applied potential can drastically alter the morphology of the precatalyst that exists under working conditions. These insights can inform future catalyst synthesis protocols, catalyst pretreatment procedures prior to operation, and novel strategies for tailoring the catalytic interfaces under operating conditions.

## Supporting Information Available

Figures S1–S12 showing additional TEM data, cyclic voltammogram from the LC-TEM experiment, and Raman spectra; Table S1 summarizing the Raman bands of metallic Bi and different phases of bismuth oxide (PDF)
