# Supplementary material for: Revealing the Intrinsic Restructuring of Bi2O3 Nanoparticles into Bi Nanosheets during Electrochemical CO2 Reduction
Source: ACS Appl Mater Interfaces. 2024 Feb 26;16(9):11552–60. doi: 10.1021/acsami.3c18285 (PMC10921375; doi:10.1021/acsami.3c18285)
Supplement: Supplementary file 1 — am3c18285_si_001.pdf [file am3c18285_si_001.pdf]

Supporting information for

Revealing the Intrinsic Re-structuring of Bi_2O_3 Nanoparticles into Bi Nanosheets during Electrochemical CO_2 Reduction

Beatriz Ávila-Bolívar*[a], Mauricio Lopez Luna[b], Fengli Yang[b], Aram Yoon[b], Vicente Montiel[a], José Solla-Gullón[a], See Wee Chee*[b], Beatriz Roldan Cuenya[b]

[a] Institute of Electrochemistry, University of Alicante, Alicante, 03690, Spain.

[b] Department of Interface Science, Fritz Haber Institute of the Max Planck Society, Berlin, 14195, Germany. E-mail:

[*] E-mail: beatriz.bolivar@ua.es, swchee@fhi-berlin.mpg.de

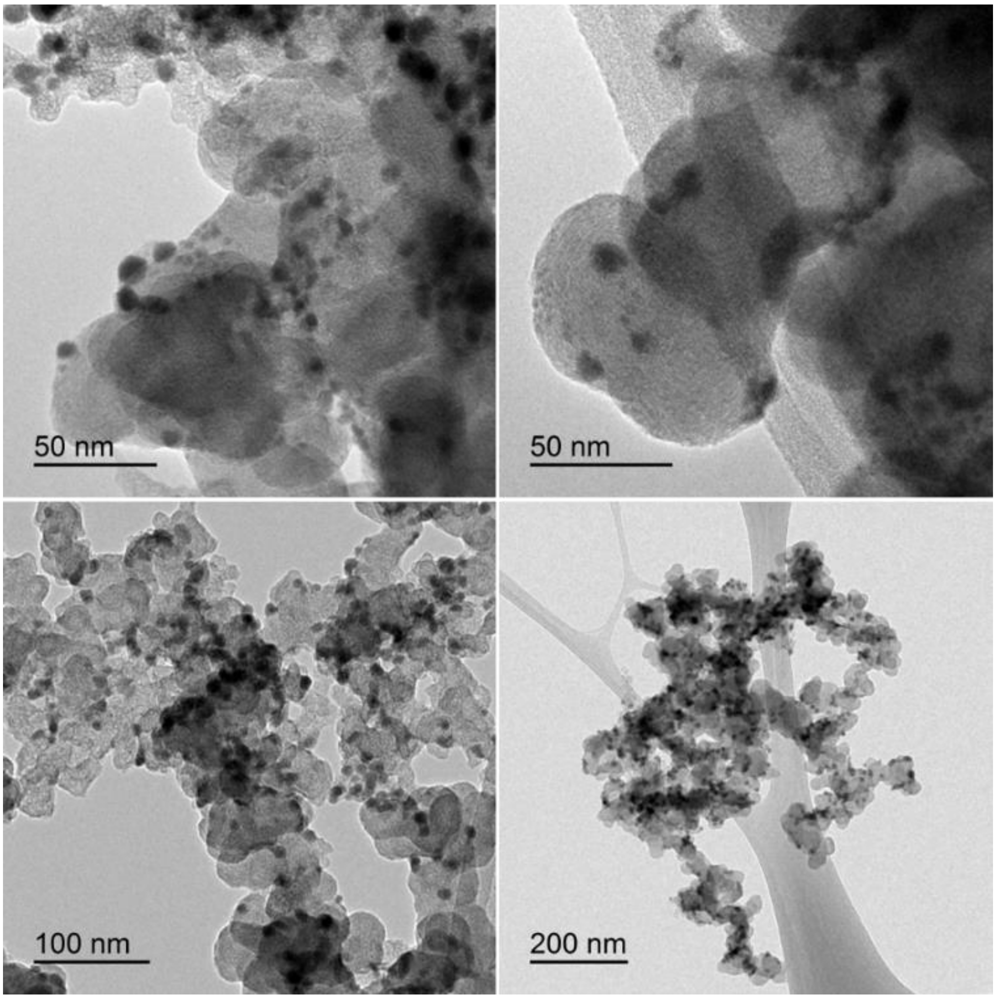

Figure S1. Representative TEM images of the Bi nanoparticles using a JEOL JEM-1400 Plus working at 120 kV.

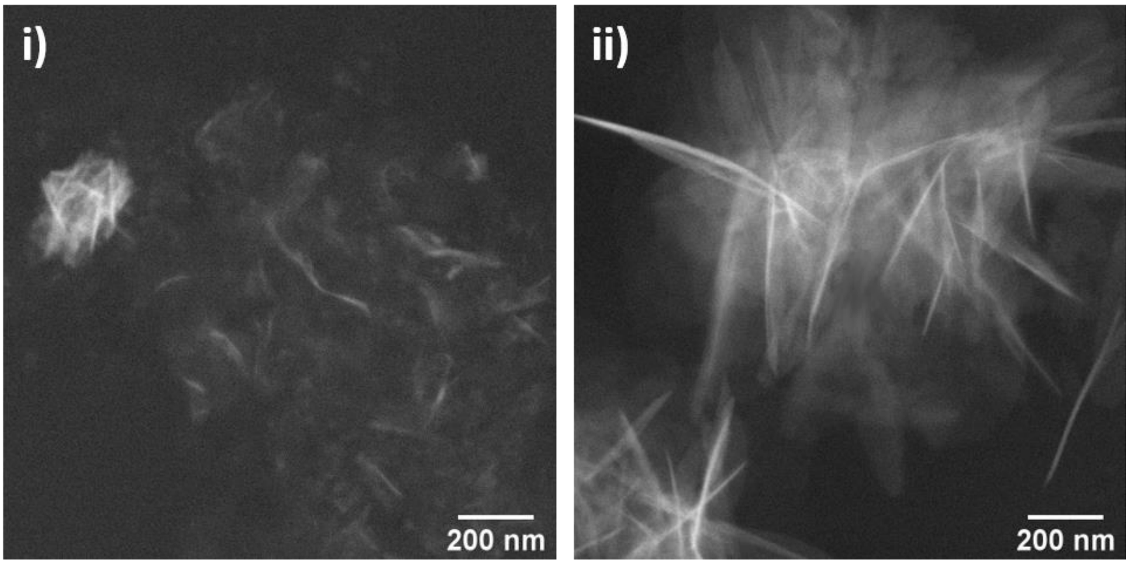

Figure S2. LC-TEM images acquired before (i) and after (ii) 0.1 M $KHCO_3$ flow. The STEM images were taken on the Titan 80-300 TEM.

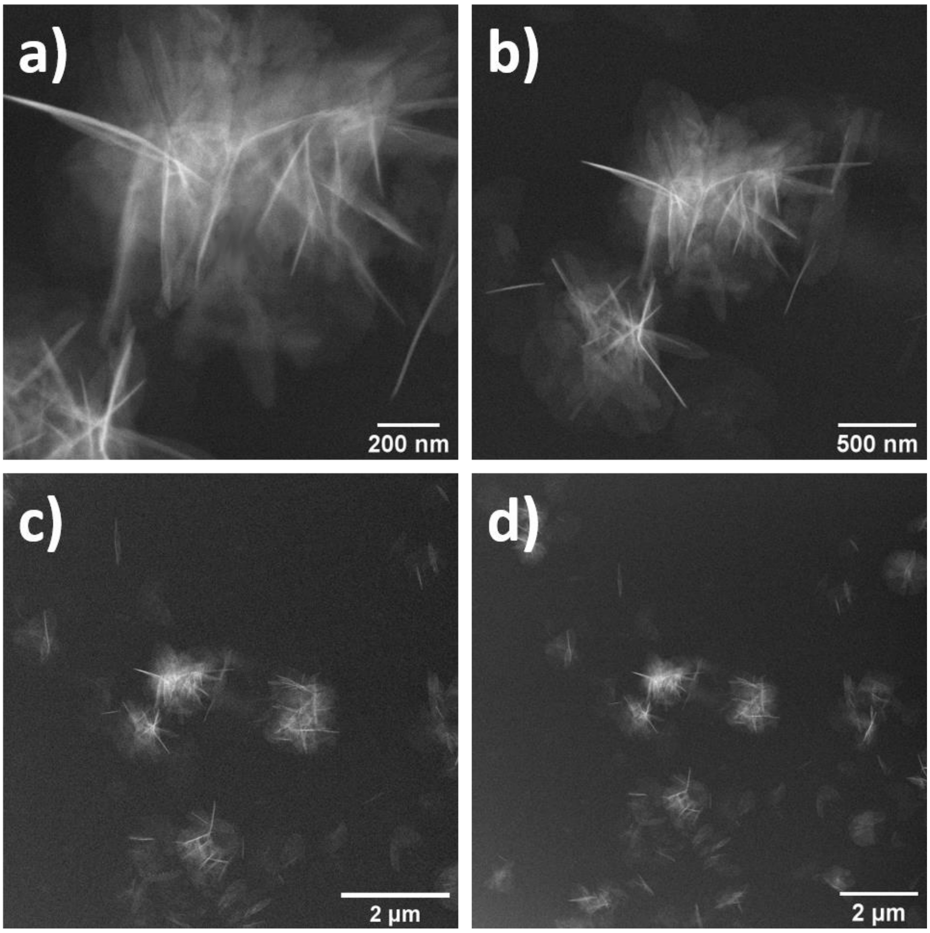

Figure S3. Final Bi structures after 10 minutes of 0.1 M $KHCO_3$ flow with decreasing magnification from (a) to (d). The STEM images were taken on the Titan 80-300 TEM.

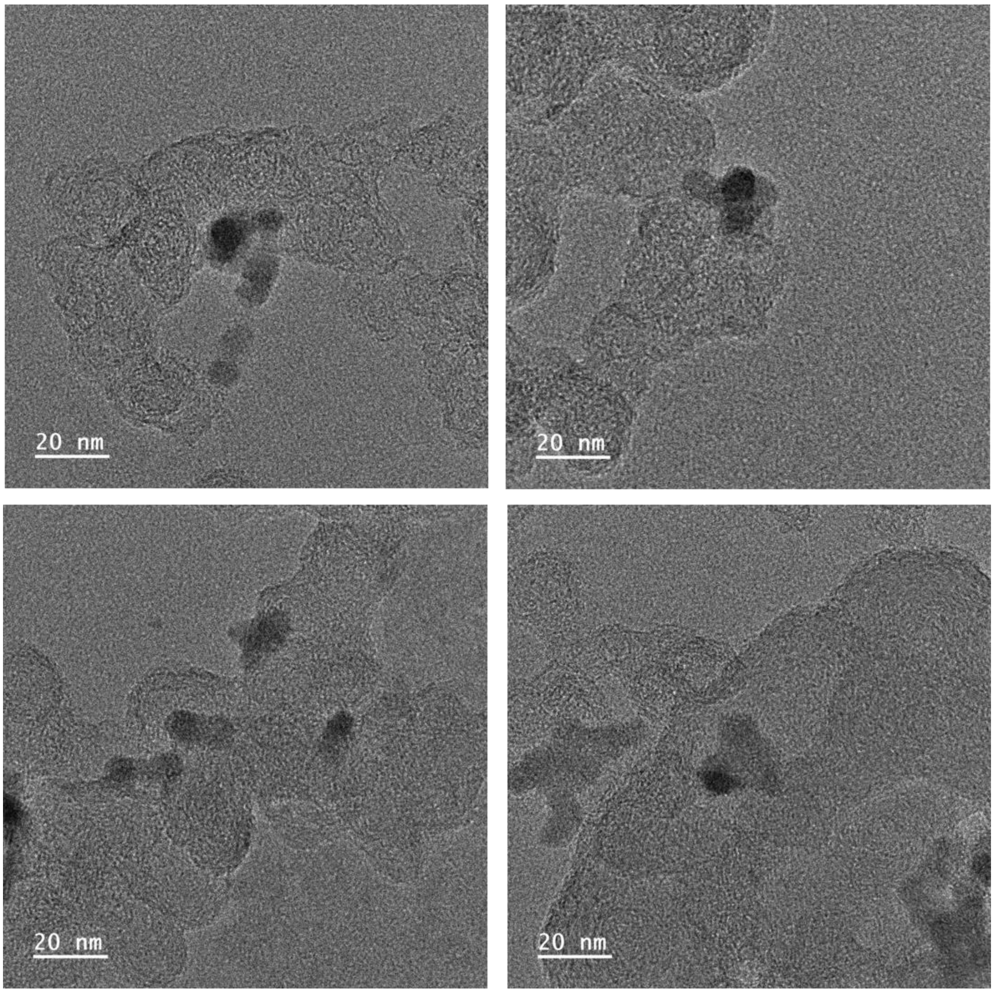

Figure S4. High resolution TEM images of the as-prepared Bi_2O_3 nanoparticles taken on the Titan microscope at 300 kV.

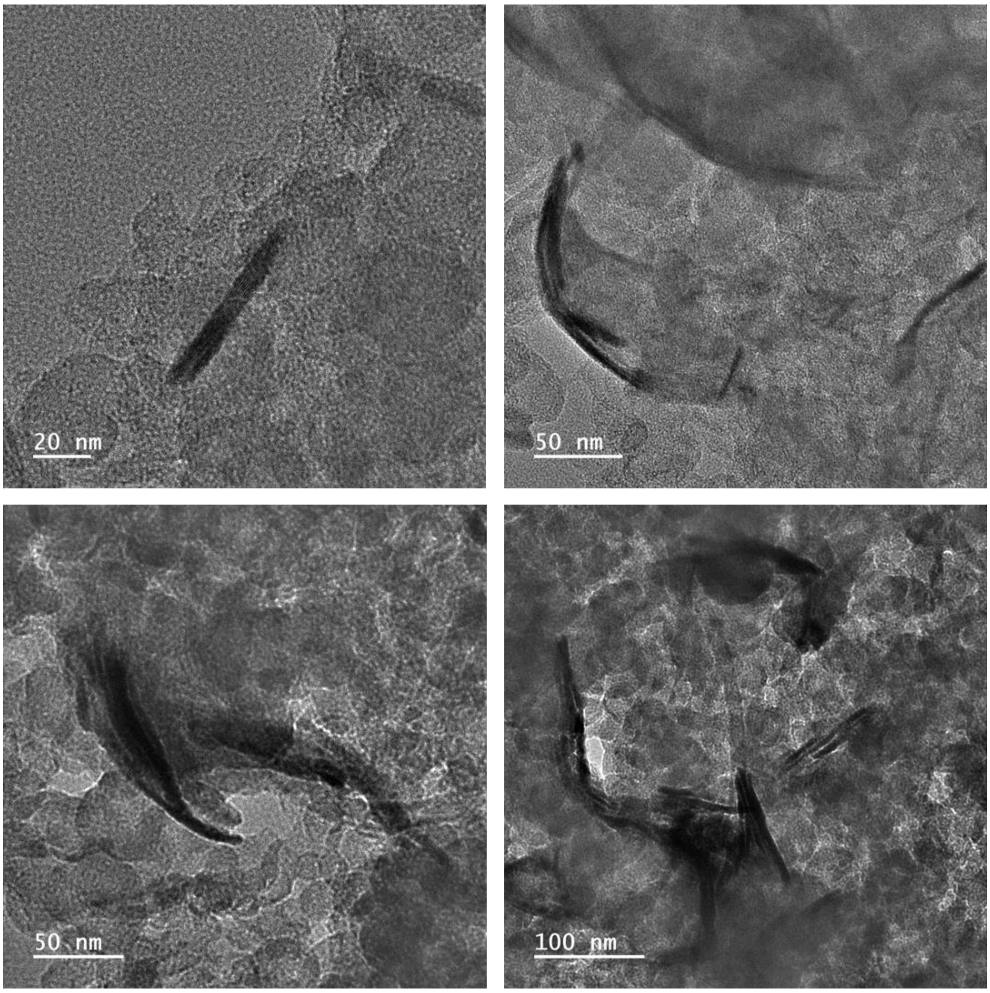

Figure S5. Hight-resolution TEM images of Bi nanosheets formed after 0.1M $KHCO_3$ flow. Taken on the Titan microscope at 300 kV.

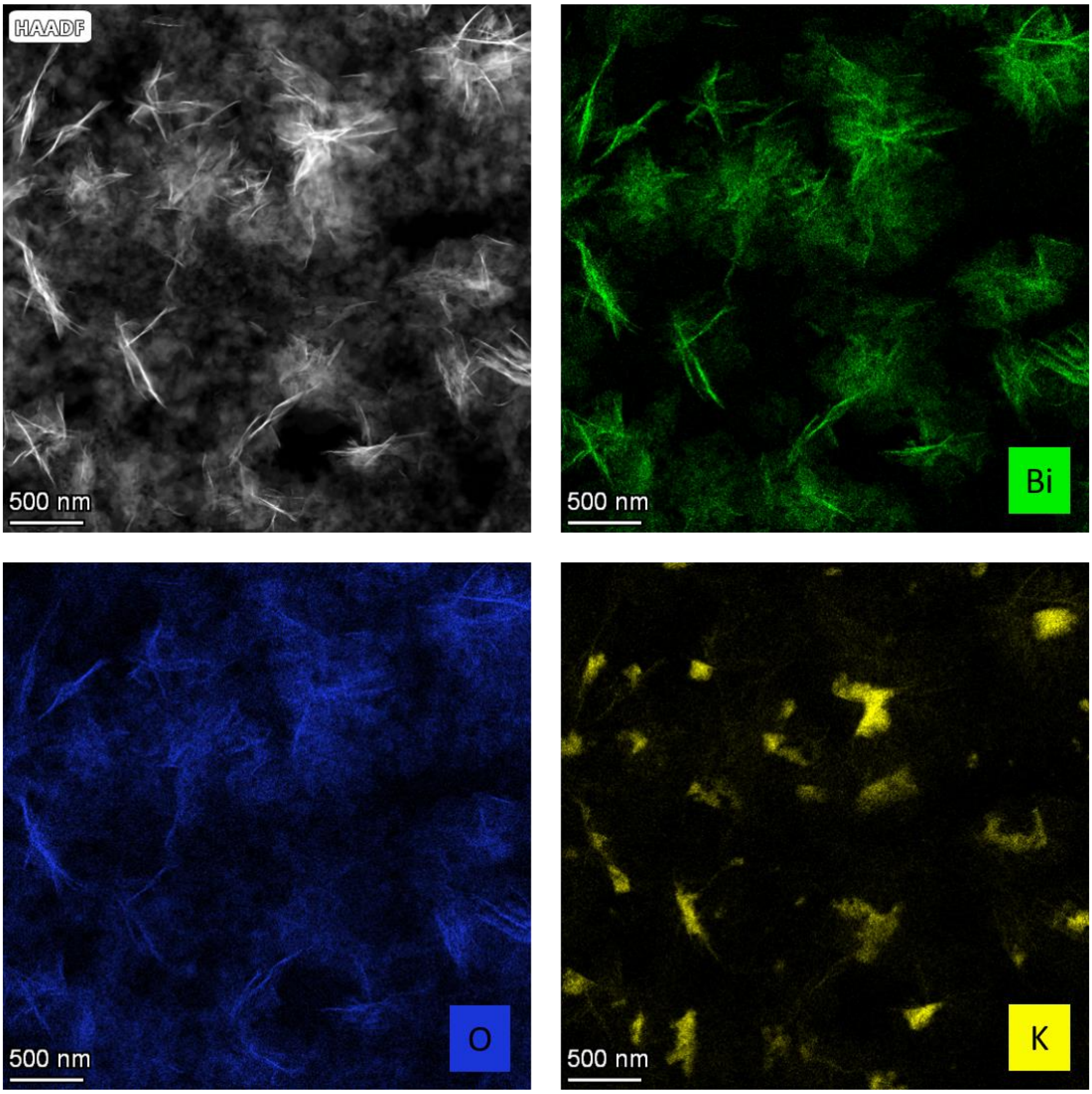

Figure S6. Ex situ EDX analysis and mapping of samples on the LC-TEM chip after 0.1M $KHCO_3$ flow using a 200 kV Talos TEM.

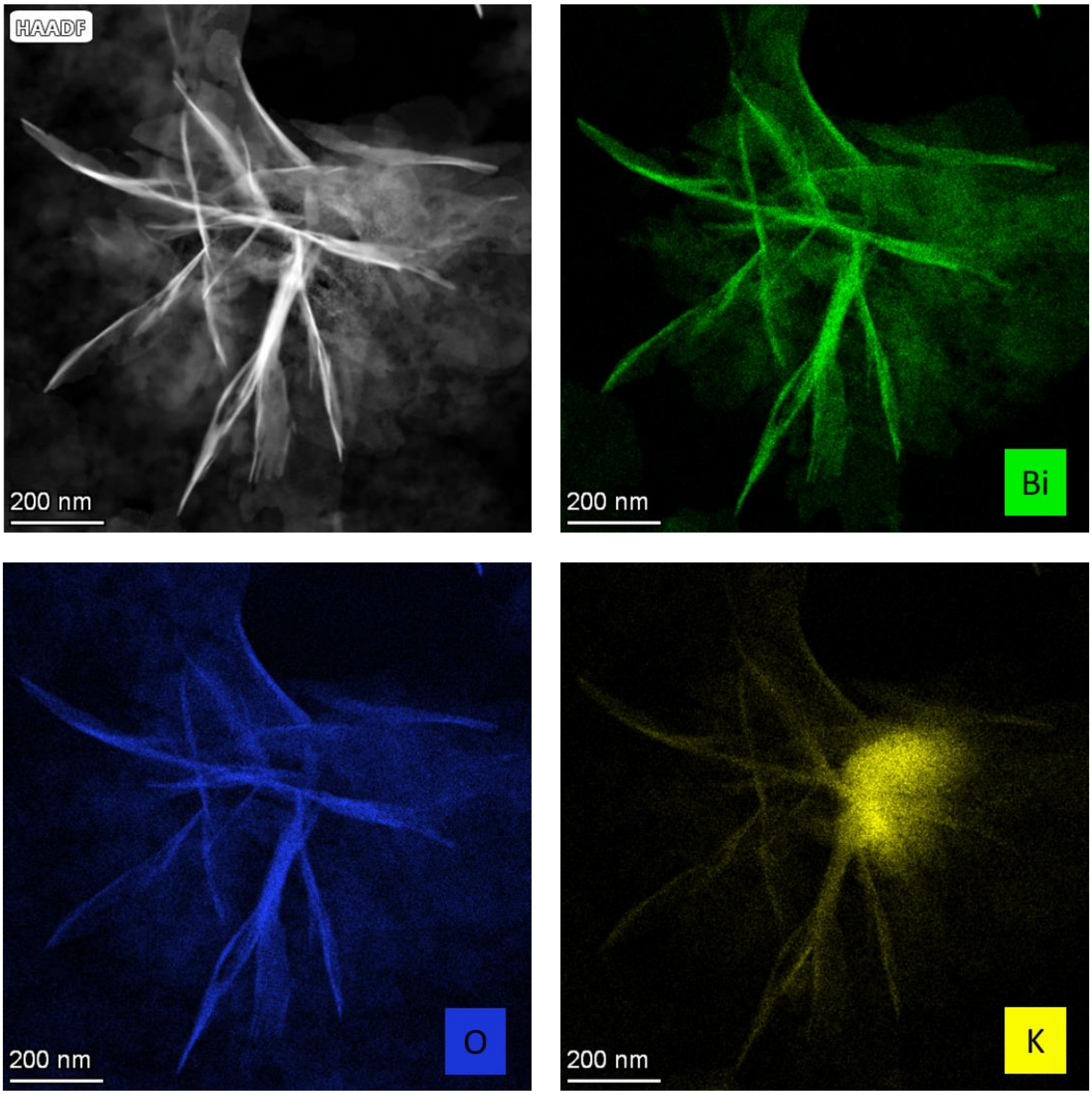

Figure S7. Ex situ EDX analysis and mapping of the chip at higher magnification after 0.1M $KHCO_3$ flow using a 200 kV Talos TEM.

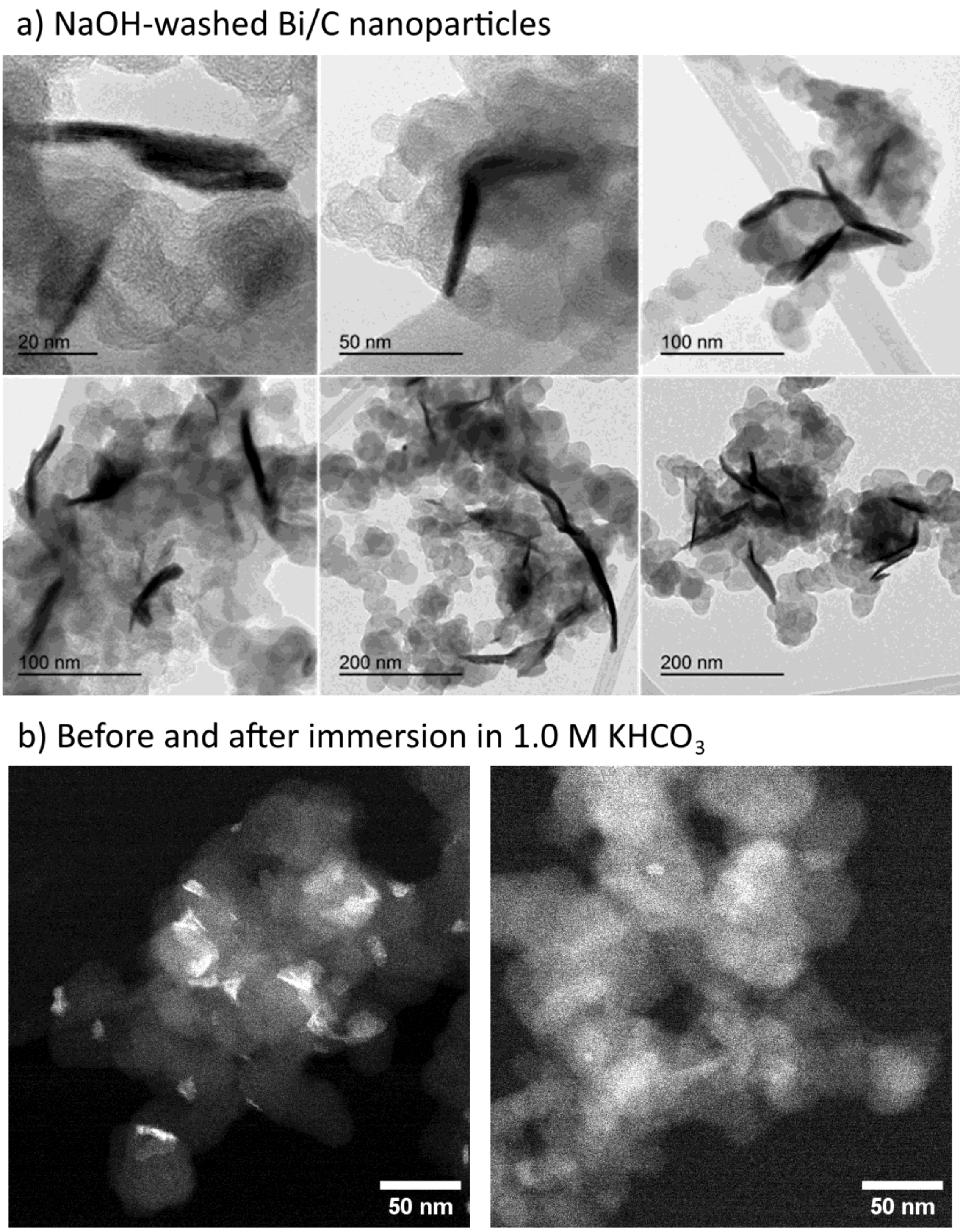

Figure S8. a) Representative TEM images of the NaOH-washed Bi/C nanoparticles using a JEOL JEM-1400 Plus working at 120 kV. b) STEM images acquired before (left) and after (right) the samples that were immersed in 1.0 M $KHCO_3$ solution for 10 minutes acquire on a Thermo Fisher Titan TEM working at 300 kV.

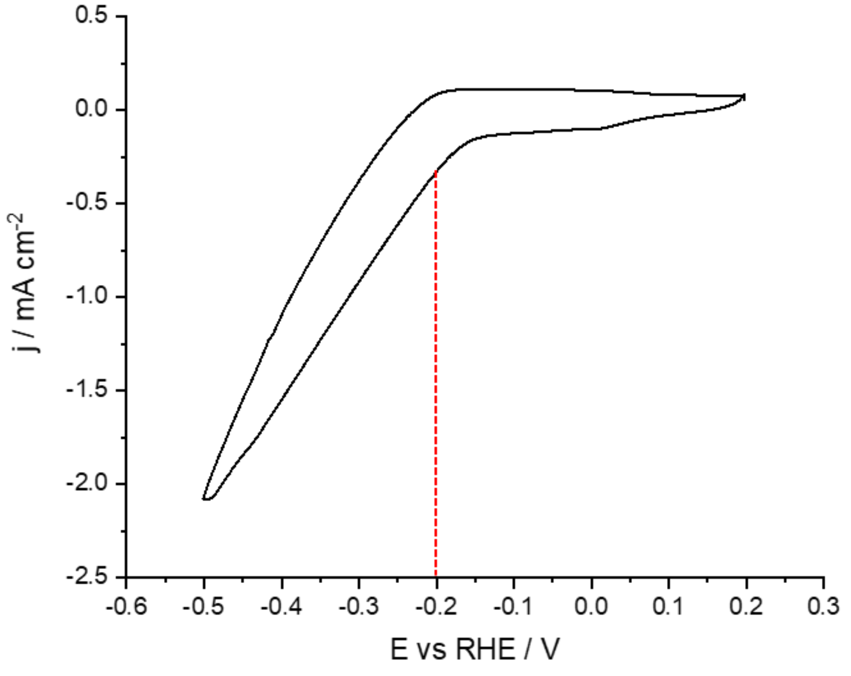

Figure S9. Cyclic voltammetry of the samples loaded on the LC-TEM chip at 50 mV s^{-1} in CO_2-saturated 0.1M $KHCO_3$ solution. The potential was measured versus an AgCl/Ag reference electrode and then converted to RHE using Nernst's equation (pH 6.8).

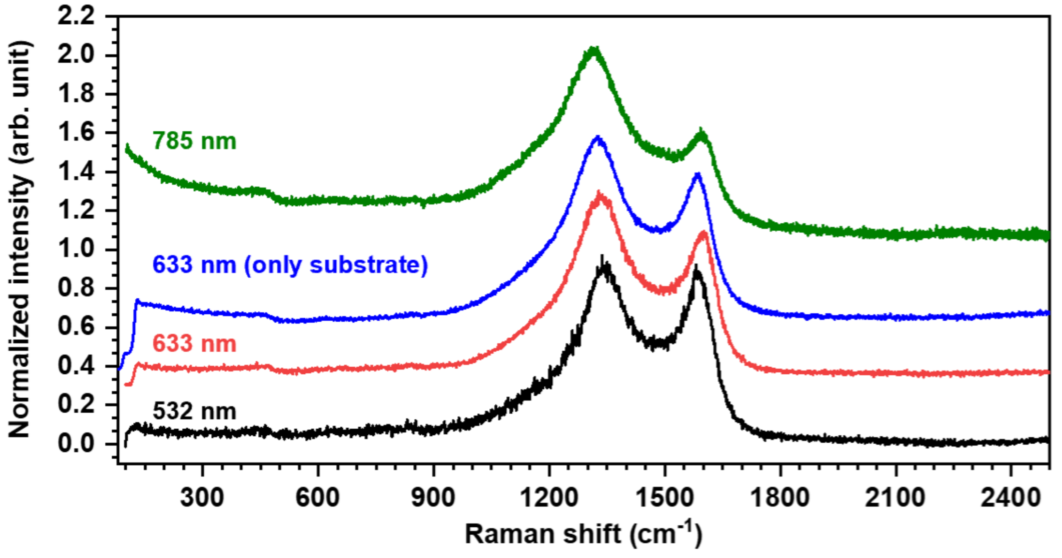

Figure S10. Raman spectra comparison of the sample under ex situ conditions (as prepared) using 3 different wavelength lasers as source of excitation.

Table S1. Raman bands reported for metallic Bi and different phases of Bismuth oxide (obtained from the references: [1–6]).

**Raman peaks (cm^{-1})**				
**Bi metallic**	**Bi_2O_3**			
	**α**	**β**	**γ**	**δ**
71	66	121-127	218	610-640
98	83	142	365	
	95	158	631	
	101	312-314	655	
	119	461-465		
	153			
	184			
	214			
	282			
	320			
	412			
	450			

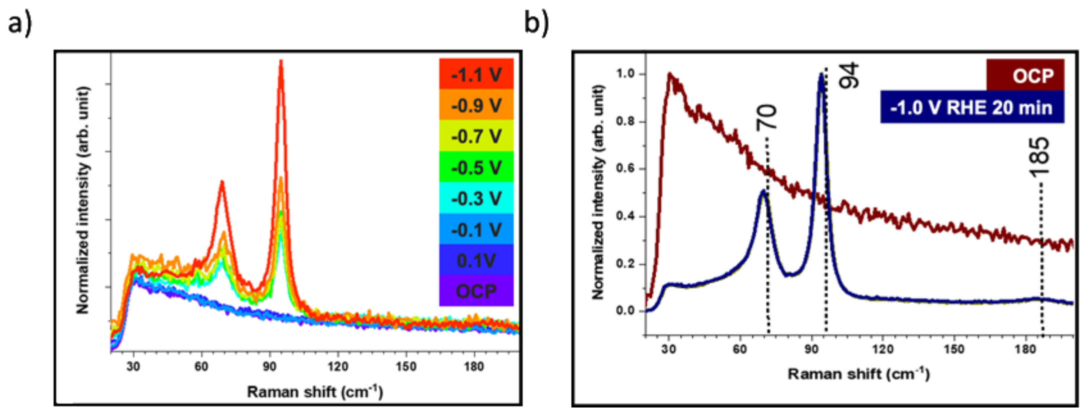

Figure S11. Operando Raman spectroscopy of the Bi/C samples collected with λ = 785 nm laser, a) at different constant potentials, and where intensity is normalized to the edge of the notch filter cut-off, and b) at OCP at t=0 and -1.0 V_{RHE} after 20 min.

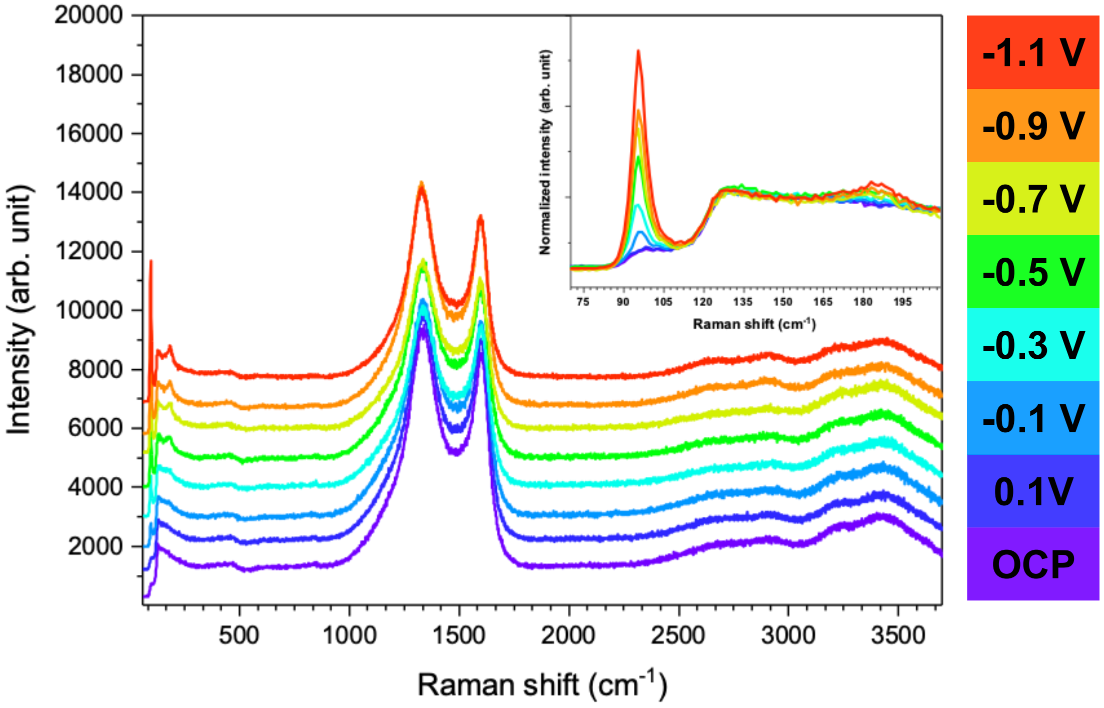

Figure S12. *Operando* Raman spectroscopy of the Bi electrode at constant stepwise potential collected with λ = 633 nm laser.

References

[1] G.S. Devi, S. V Manorama, V.J. Rao, SnO2 / Bi2 O 3: A Suitable System for Selective Carbon Monoxide Detection, J. Electrochem. Soc. 145 (1998) 1039. https://doi.org/10.1149/1.1838385.

[2] H.T. Fan, S.S. Pan, X.M. Teng, C. Ye, G.H. Li, Structure and thermal stability of δ-Bi2O3 thin films deposited by reactive sputtering, J. Phys. D. Appl. Phys. 39 (2006) 1939. https://doi.org/10.1088/0022-3727/39/9/032.

[3] M. Vila, C. Díaz-Guerra, J. Piqueras, Laser irradiation-induced α to δ phase transformation in Bi2O3 ceramics and nanowires, Appl. Phys. Lett. 101 (2012) 71905. https://doi.org/10.1063/1.4747198.

[4] J.A. Steele, R.A. Lewis, In situ micro-Raman studies of laser-induced bismuth oxidation reveals metastability of β-Bi2O3 microislands, Opt. Mater. Express. 4 (2014) 2133–2142. https://doi.org/10.1364/OME.4.002133.

[5] S. Kim, W.J. Dong, S. Gim, W. Sohn, J.Y. Park, C.J. Yoo, H.W. Jang, J.-L. Lee, Shape-controlled bismuth nanoflakes as highly selective catalysts for electrochemical carbon dioxide reduction to formate, Nano Energy. 39 (2017) 44–52. https://doi.org/https://doi.org/10.1016/j.nanoen.2017.05.065.

[6] H. Wang, C. Tang, B. Sun, J. Liu, Y. Xia, W. Li, C. Jiang, D. He, X. Xiao, In-situ structural evolution of Bi_2O_3 nanoparticle catalysts for CO_2 electroreduction, Int. J. Extrem. Manuf. 4 (2022) 35002. https://doi.org/10.1088/2631-7990/ac7a6e.
